# Supplementary material for: SARS-CoV-2 Omicron Variant, Lineage BA.1, Is Associated with Lower Viral Load in Nasopharyngeal Samples Compared to Delta Variant
Source: Viruses. 2022 Apr 28;14(5):919. doi: 10.3390/v14050919 (PMC9144383; doi:10.3390/v14050919)
Supplement: Supplementary file 1 [file viruses-14-00919-s001.zip › viruses-1654916-supplementary.pdf]

Supplementary 1

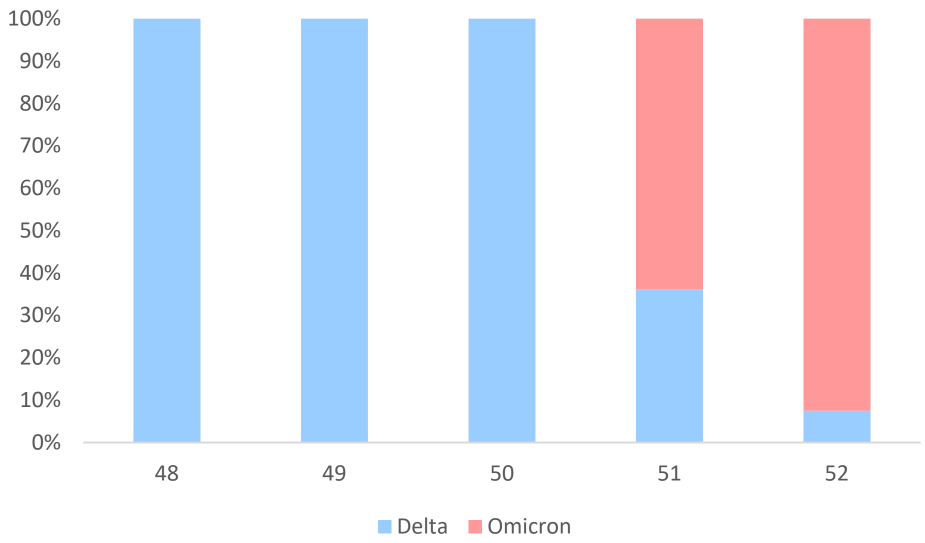

Figure S1. Proportion of samples positive for the Delta or Omicron variant on a weekly basis. Week 48 (Delta, n=28), week 49 (Delta, n=16), week 50 (Delta, n=16); week 51 (Delta, n= 17; Omicron, n=30), week 52 (Delta, n= 8; Omicron, n=99).

The appendix is an optional section that can contain details and data supplemental to the main text—for example, explanations of experimental details that would disrupt the flow of the main text but nonetheless remain crucial to understanding and reproducing the research shown; figures of replicates for experiments of which representative data is shown in the main text can be added here if brief, or as Supplementary data. Mathematical proofs of results not central to the paper can be added as an appendix.

Supplementary 2

	Delta		Omicron		
**RT-PCR SARS-CoV-2 COBAS**	**Ct**	**SD**	**Ct**	**SD**	**p-value**
All samples	22.62	4.39	24.4	4.38	**0.004**
**Symptoms**					
Asymptomatic prior samples	24.06	4.81	25.62	4.42	0.19
Symptoms <5 days prior samples	21.7	4.02	23.79	4.47	**0.008**
Symptoms day before or day of sample	21.35	3.85	24.19	5.31	**0.035**
Symptoms 2 to 4 days before sample	21.94	4.18	23.38	3.46	0.13
Symptoms >5 days prior sample	23.69	4.07	24.61	3.71	0.6
**Vaccination**					
Complete vaccination	21.85	4.26	24.86	4.41	**0.001**
Boosted vaccination	25.79	4.11	23.84	4.21	0.08
**Sex**					
Women	22.61	4.48	24.37	4.22	**0.02**
Men	22.63	4.29	24.46	4.69	0.09
**Age**					
< 30 years old	23.49	4.50	24.77	4.67	0.21
31-40 years old	23.11	4.75	24.77	4.38	0.16

41 -50 years old	20.53	2.28	23.32	3.64	**0.01**
> 51 years old	21.34	4.53	23.93	4.35	0.16
≤40 years old	23.31	4.58	24.77	4.55	0.055
>40 years old	20.87	3.34	23.56	3.88	**0.006**

Supplementary Table S1: Cycle threshold analysis between Delta and Omicron variant according to symptoms, vaccination status, sex and age. P-value was calculated with a Student test.

Supplementary 3

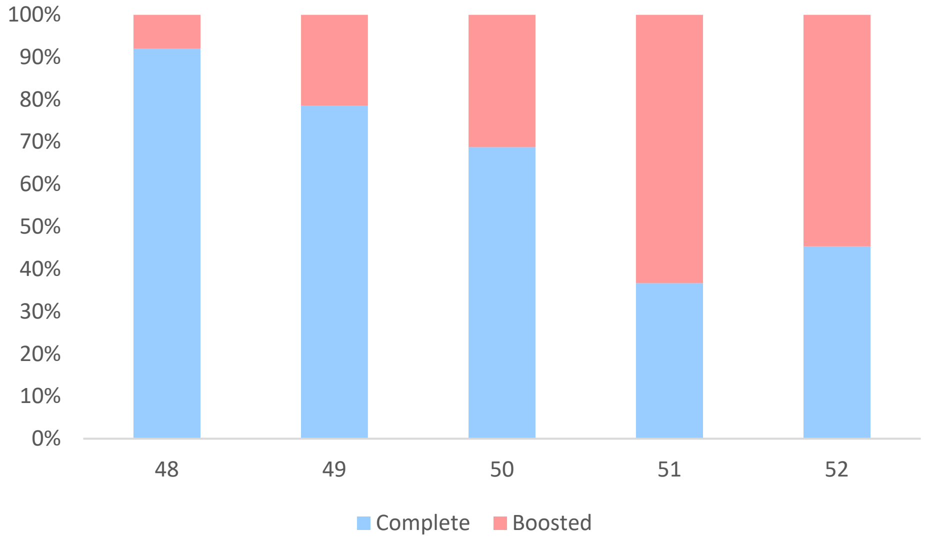

Supplementary Figure S2: Vaccination status of patients on a weekly basis. Patients not vaccinated, partially vaccinated, or with unknown status were removed from this graph. Week 48 (complete, n= 23: boosted, n = 2), week 49 (complete, n =11; boosted, n =3), week 50 (complete, n= 11; boosted, n =5), week 51 (complete, n = 11; boosted, n = 19), week 52 (complete, n = 39; boosted, n= 47).
